# A meta-analysis on the relationship between subjective cognitive failures as measured by the cognitive failures questionnaire (CFQ) and objective performance on executive function tasks

**DOI:** 10.3758/s13423-024-02573-6

**Published:** 2024-09-09

**Authors:** Stephanie C. Goodhew, Mark Edwards

**Affiliations:** School of Medicine and Psychology, The Australian National University, Canberra, Australia

**Keywords:** Cognitive failures, Cognitive Failures Questionnaire, Executive function, Working memory, SART, Inhibition, Shifting, Switching

## Abstract

The Cognitive Failures Questionnaire (CFQ) has been widely used as a measure of subjective cognitive function in everyday life for decades. However, the evidence on how it relates to objective performance on executive function tasks is mixed. One possible reason for these mixed results is that the CFQ has selective relationships with some aspects of executive function and not others. Here, therefore, we classified tasks according to an influential framework of executive functions—switching, updating, inhibition, and we also considered the Sustained Attention to Response Task (SART) as a category because it was custom designed to gauge cognitive failures. We synthesized a large body of available evidence and performed four Bayesian meta-analyses on the relationship between CFQ scores and objective performance on executive function tasks in these four categories. Results suggested that CFQ scores were associated with objective performance on SART (18 effect sizes, μ = −.19, BF_10_ = 18.03, i.e., 18.03 times more evidence of a relationship versus no relationship), updating working memory (49 effect sizes, μ = −.06, BF_10_ = 17.80), and inhibition tasks (41 effect sizes, μ = −.07, BF_10_ = 15.40), whereas there was not definitive evidence regarding switching (34 effect sizes, μ = −.06, BF_10_ = .50, i.e., two times greater evidence for no relationship). This suggests that subjective cognitive function can predict objective performance on at least some executive function tasks. We discuss methodological and theoretical factors that constrain the maximum observable correlation and consider the relative insights that subjective measures versus task performance provide.

**Supplementary Information:**

The online version contains supplementary material available at 10.3758/s13423-024-02573-6.

## Introduction

Cognitive failures—such as forgetting appointments, daydreaming when you ought to be listening, and forgetting what you came to the shops to buy—are common human experiences. However, people differ in the frequency with which they experience these cognitive failures, and these differences are measured by the Cognitive Failures Questionnaire (CFQ; Broadbent et al., 1982). The CFQ has widespread currency in experimental, clinical, and applied work, as reflected in the fact that the paper introducing the CFQ has been cited more than 3,500 times at the time of this writing. The CFQ scores have been found to be associated with meaningful outcomes, such as that those who experience greater cognitive failures are more prone to work accidents and car accidents (Larson et al., 1997; Wallace & Vodanovich, 2003) and have impaired work ability (Gajewski et al., 2023). They also show more difficulty regulating their emotions (Robins et al., 2012) and have lower life satisfaction (Haslam et al., 2008).

Some studies have found that those who experience greater cognitive failures perform worse on tasks that measure attentional control or executive functioning (e.g., Forster & Lavie, 2007; Friedman & Miyake, 2004; Goodhew & Edwards, 2023; Manly et al., 1999; McVay & Kane, 2009). However, not all studies find such relationships (e.g., Carrigan & Barkus, 2016; Keilp et al., 2013; Richter et al., 2015; Ryskin et al., 2015). Some of this variance may be due to the specific type of task used. For example, studies that measure performance on the Sustained Attention to Response Task (SART) appear to find relationships with CFQ scores reasonably consistently (e.g., Linden et al., 2005; Manly et al., 1999; Robertson et al., 1997; Smilek et al., 2010). In contrast, for other tasks, such as Stroop, the findings are more mixed, with some studies finding a relationship between performance and CFQ scores (e.g., Adjorlolo, 2016; Vom Hofe et al., 1998) and others not (e.g., Gajewski et al., 2011; Gunduz et al., 2022; Keilp et al., 2013). Similarly, some studies find a relationship between performance on the Trail Making Test B and CFQ scores (e.g., Gajewski et al., 2011; Miskowiak et al., 2023), whereas others do not (e.g., Baker et al., 2018; Bellens et al., 2022). These differences could reflect the CFQ having selectively stronger associations with some aspects of executive function than others. Further, some studies have found no relationship between CFQ scores and memory when it is measured via tasks that simply require the retention of information (e.g., Castellon et al., 2004; Rodgers, 2000). But retention of information is not an executive function, whereas *updating working memory—*which requires the processing and updating of information in memory—is an executive function, and tasks that gauge updating working memory have been found to correlate with CFQ scores (e.g., Chang et al., 2021; but see Ryskin et al., 2015). However, attempting to draw such conclusions from isolated comparisons is fraught because these studies all differ in other ways which could have affected the outcomes, such as the number of participants in the study. Further, evidence for small effects may only be revealed when data are collated across multiple studies. This is where meta-analysis is a particularly powerful and useful tool, because it can synthesize a large body of available findings to assess whether there is overall evidence for an effect, and whether this depends on factors such as task type.

Here, therefore, we performed meta-analyses on the large body of available evidence on the relationship between CFQ scores and objective performance on executive function tasks to assess whether there is a robust relationship, and whether this differed according to different aspects of executive function. In particular, we applied an influential model of executive functions that identifies switching,^1^ working memory updating, and inhibition, as three distinct components of executive functions (Miyake et al., 2000). In addition, we considered the association between CFQ scores and performance on a particular task—the SART—because this task was specifically developed to gauge the same type of cognitive errors as gauged by the CFQ (Robertson et al., 1997). That is, in the SART, a dominant response set is developed by responding with a particular key press to most stimuli, but then having to withhold a response when an infrequently occurring stimulus appears. Errors of commission—responding when a response should be withheld—are thought to reflect the same type of everyday cognitive lapses captured by the CFQ (Manly et al., 1999; Robertson et al., 1997). Therefore, we measured the strength of the relationship between CFQ scores and objective performance where cognitive tasks were classified into one of four categories: switching tasks, working memory updating tasks, inhibition tasks, and SART.

## Method

**Fig. 1:**
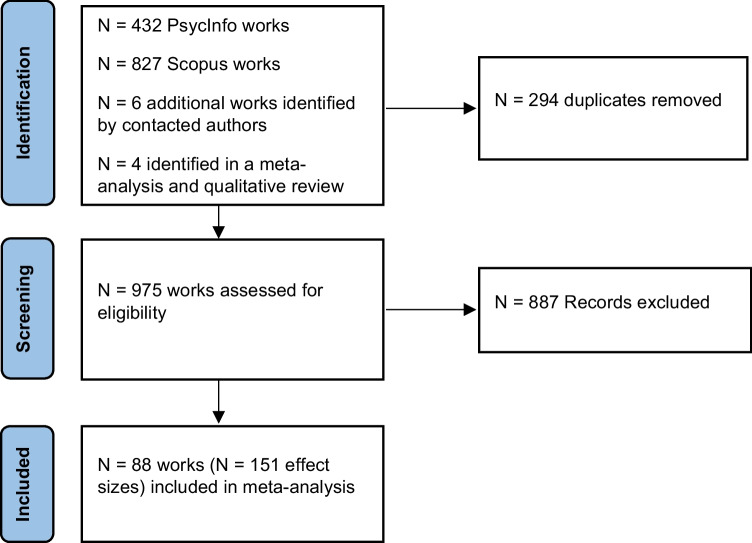
A flow chart of the identification and screening process

The search for relevant works was conducted on 7 July 2023. Two databases were used: PsycInfo and Scopus. In PsycInfo, title, abstract, and key concepts were searched with the following terms: (‘cognitive failures’ OR ‘CFQ’ OR ‘subjective cogniti*’) AND (‘executive function’ OR ‘working memory’ OR ‘inhibition’ OR ‘inhibitory control’ OR ‘shifting’ OR ‘switching’ OR ‘updating’ OR ‘SART’ OR ‘sustained attention’). This yielded 432 results. In Scopus, title, abstract, and keywords were searched with the same terms as PsycInfo, which yielded 827 results. This means that a total of 1,259 works were initially identified (see Fig. 1). From these, there were 965 unique works after duplicates were removed. In the process of assessing these 965 for eligibility, an additional 10 works were identified (six from authors and four from additional references), as described below. This means that a total of 975 works were ultimately assessed for eligibility.

For studies that contained both CFQ and one or more eligible performance measures, but did not report the correlation, the corresponding author was emailed on the address listed on the paper to ask for this information. If this did not elicit a response, then a search was done to attempt to identify their current email address, and they were contacted on this if available, and/or the current address of other authors. In addition, corresponding authors of recent works (published from 2014 onwards) who reported the relevant correlation(s) in their work were contacted see if they had other published or unpublished work that would meet eligibility criteria. An additional six works were identified this way (of which four met eligibility criteria). In addition, an older meta-analysis on CFQ–SART associations (Smilek et al., 2010) and a qualitative review that treated cognitive function as unitary (Carrigan & Barkus, 2016) were identified amongst the *N* = 965 records. While these reviews were not eligible (although Smilek et al., 2010, also included an original experiment that was), their identified references were checked against the 965 records. From this, four additional older works that were not identified in the original search were identified and all assessed as eligible for inclusion. A flow chart of this process is shown in Fig. 1.

The eligibility criteria were as follows:

1. The work must be an original study (as opposed to a meta-analysis or review), but could be published or unpublished, and could be a thesis.
2. The sample size must be greater than 1 (i.e., case studies not included).
3. If the same data were presented in a thesis and a published paper, then only the published paper was counted as eligible.
4. The study must be with adults and could include clinical groups. This is because correlations can be constrained if there is insufficient range in a variable such as cognitive failures. Excluding clinical groups (e.g., those with ADHD) could unduly constrain the range of CFQ scores.
5. CFQ scores must be provided by the person performing the objective task (i.e., self-rated CFQ scores). This is because we were interested in the CFQ as a measure of an individual’s subjective cognitive function.
6. While most studies used the original 25-item CFQ, other variants of the CFQ were eligible if they included original items from the CFQ. The role of CFQ type was assessed via statistical analysis.
7. The study must report an association (or the data were available so that an association could be calculated, or authors provide the association on request) between CFQ scores and objective performance scores on an eligible task (see below). While correlation coefficients were preferred, regression coefficients were included if that was all that was available, as were tests of participants categorised as low versus high scorers on the CFQ.
8. Where a study explicitly only reported significant correlation coefficients and omitted nonsignificant correlation coefficients, if the nonsignificant correlation coefficients could not be obtained on request, then these significant coefficients were *not* included so as not to introduce bias toward significant results (two studies).
9. If the study was assessing an intervention, then correlation(s) between CFQ and performance that were assessed at baseline (preintervention) only were eligible.
10. Where two works used the same participants (e.g., a later follow-up), the earlier study only was considered eligible because this would have the maximum *N* size (smaller at follow-up due to drop-out).
11. Where a work had multiple potential relevant measures in a single category (e.g., three different measures of updating working memory), then the average effect sizes for each of these measures was computed, unless the authors had already computed a composite of the relevant measures (Lange & Süß, 2014; McVay & Kane, 2009; Unsworth et al., 2012), in which case this was used.
12. Where a work had eligible effect sizes in different categories (e.g., a measure of updating working memory and a measure of inhibition), all eligible effect sizes were included (i.e., a single study could contribute effect sizes to multiple categories).

Where studies reported correlations for separate groups (e.g., patients vs. controls), or multiple experiments with different participants, these were treated as separate effect sizes. Since these are with different samples, this does not undermine the independence of effect sizes. However, it was also common for a given study to contribute multiple effect sizes in different categories (see eligibility criterion #12). This is because many studies had participants complete a battery of executive function tasks in addition to the CFQ. Had we restricted each study to only contributing a single effect size, then we would have had to make arbitrary choices about which to include, and we would have been discarding large amounts of data. We reasoned that it was better to use all this available data. However, consequently, the effect sizes in different categories are not truly independent of one another. For example, the same participants may have contributed an effect size to the switching and inhibition categories. For this reason, we performed a meta-analysis separately on each of the four categories of executive function tasks (i.e., switching, updating working memory, inhibition, SART).

For Category 1, we identified tasks that operationalise *switching* of task sets or goals (see Table 1). This includes the classic Wisconsin Card Sorting Test (WCST; Berg, 1948), which measures the switching component of executive function (Miyake et al., 2000). The preferred metric was perseverative errors, but if unavailable then overall accuracy metrics were used. The intra-extra dimensional set shift offered in CANTAB® (Cambridge Cognition) was considered equivalent to the WCST, although we were not able to obtain any the required correlation coefficients for studies that used this measure, and we have therefore omitted it from Table 1. Trail Making Test B (Reitan, 1955), where participants have to switch between using letters and numbers to determine where to draw a line, also measures switching and was therefore eligible (Arbuthnott & Frank, 2000). Trail Making Test B scores reflect the time taken to complete the task. Other task-switching paradigms were also eligible, which were defined as where participants were performing one task (e.g., classifying digits as odd/even) and then must switch to a new task (e.g., classifying digits as larger or smaller than 5). Switch cost or absolute accuracy and/or RT in switch conditions (used whichever available, or average of accuracy and RT if both available).

**Table 1:** Main eligible tasks for each category of executive function

Category	Task	Description
1	Wisconsin Card Sorting Test	Match cards according to dimensions (e.g., colour, shape) where correct dimension shifts
1	Trail Making Test B (or D-KEFS Condition 4)	Draw a line between consecutive circles where alternate between numbers and letters
1	Task switching	Where performing one task (e.g., classifying digits as odd/even) and then must switch to a new task (e.g., classifying digits as larger or smaller than 5)
2	Reading span	Remembering an updating series of letters whose presentation is interspersed with reading and assessing sentences
2	Operation span	Remembering an updating series of letters whose presentation is interspersed with performing mental arithmetic to assess math problems
2	Symmetry span	Remembering an updating series of spatial locations in a matrix whose presentation is interspersed with making symmetry judgements
2	Digit span backwards	Presented with digits to remember and must report them in reverse order to presentation
2	Digit span sequencing	Presented with digits and then must report them in numeric sequential order
2	Letter number sequencing	Presented with letters and numbers, and then recall letters alphabetically and numbers sequentially
2	Corsi block tapping reverse or backward	Blocks positioned on a board, a series of blocks are tapped or presented, and participants repeat the sequence of blocks in reverse order
2	Tasks that require the reversal or other changed sequencing of to-be-remembered information	For example, presenting a series of stimuli and participants are required to report them in reverse order
2	*n*-back	Presented with a sequence of stimuli, and the task is to indicate when the current stimulus matches the one from *n* steps earlier in the sequence
2	PASAT and PVST	Presented with numbers and have to add the number seen/heard with number they saw/heard before
3	Stroop task or Colour-Word Interference III (D-KEFS)	Task is to name colour of word where there is a conflict between colour and meaning of word (e.g., RED in blue)
3	Flanker task	Task is to identify a target with conflicting stimuli presented at same time as target
3	Simon task	Task is to identify a target when stimulus and response are on opposite sides
3	Antisaccade task	Task is to saccade in opposite direction to a salient stimulus
3	Go/no-go task	Respond on certain trials (‘go’ trials) while withholding response on other trials (‘no-go’)
3	Stop-signal task	Variant of go/no-go, except stop-signal, which instructs participants not to respond, occurs after presentation of go stimulus
3	Response conflict tasks	Any task where one had to respond to targets and concurrent nontargets could produce interference
4	SART	Respond to most stimuli with a particular key; have to withhold response to a given infrequent stimulus

*Note.* D-KEFS = Delis-Kaplan Executive Function System^TM^; PASAT = Paced Auditory Serial Addition Test; PVST = Paced Visual Serial Addition Task; SART = Sustained Attention to Response Task

For Category 2, we included tasks that measured *working* memory capacity, as opposed to mere retention of information. That is, only tasks that required participants to update and/or perform some type of mental manipulation of the to-be-remembered material were included. Tasks that simply required the retention of information were not eligible. Eligible tasks included reading span, operation span (OSPAN), and symmetry span tasks (Conway et al., 2005; Daneman & Carpenter, 1980; Redick et al., 2012; Shah & Miyake, 1996; Turner & Engle, 1989). These are complex span tasks that require participants to juggle two tasks and update the list of to-be-remembered material, and such tasks have been linked to the *updating* component of working memory (Miyake et al., 2000). The complex span used in one study (Guye et al., 2020) was considered equivalent to symmetry span because it involved memorising the positions of red squares presented in a grid, where the presentation of the squares was interleaved by another task (judging orientation of *T*s and *L*s). The computation span used in one study (Montgomery & Fisk, 2007) was considered equivalent to OSPAN because participants were solving arithmetic problems while also simultaneously remembering the second digit of each presented problem. Dot span was also considered eligible as a complex span when it was aggregated with reading span, operation span, and other updating tasks (Lange & Süß, 2014).

Digit span backwards (rather than forwards) has been linked to working memory (Hale et al., 2002; Reynolds, 1997; Yoshimura et al., 2023), and digit span sequencing may be especially linked to working memory (Meyer & Reynolds, 2018; Werheid et al., 2002). Similarly, there is evidence that letter–number sequencing reflects working memory (Crowe, 2000; Haut et al., 2000). Therefore, digit span backwards, digit span sequencing, and letter–number sequencing were eligible for Category 2, whereas digit span forwards was not. In a similar vein, tasks that required participants reverse or otherwise change the sequence of remembered information were considered eligible, such as the Corsi block tapping reverse (Ahmad et al., 2022; Könen & Karbach, 2020), visual working memory backwards (Kitahata et al., 2017), and dot matrix and spatial span backwards (Peers et al., 2022).

The *n*-back requires online processing and updating of information and was therefore considered eligible for Category 2. Although there have been some questions about its relationship with other measures of working memory (e.g., Jaeggi et al., 2010; Redick & Lindsey, 2013), it has been suggested to capture working memory function and to detect subtle changes in cognitive function that other tasks were not able to detect (Miller et al., 2009), and therefore it was considered eligible. Where more than one *n* was available (e.g., 2-back and 1-back), the highest *n* available was used to obtain the relevant effect size. Further, the Paced Auditory Serial Addition Test (PASAT; Bloomfield et al., 2010; Nikravesh et al., 2017), and its visual counterpart, the Paced Visual Serial Addition Task (PVST; Krabbe et al., 2017; Nagels et al., 2005), require online mental arithmetic with the to-be-remembered information, and were therefore considered eligible for Category 2.

For Category 3, tasks that gauge *inhibition* were eligible. Specifically, tasks that require inhibiting salient information including Stroop (MacLeod, 1991; Stroop, 1935), Simon (Hommel, 2011; Simon & Rudell, 1967), flanker (Burgoyne et al., 2023; Eriksen & Eriksen, 1974), and antisaccade (Everling & Fischer, 1998; Hutton & Ettinger, 2006) were eligible for Category 3. Any other task where participants were responding to targets and concurrent nontargets could produce interference were also eligible. For all, the preferred measure was interference (i.e., difference in performance between incongruent and congruent conditions), but if unavailable, then performance in the incongruent condition itself was used. Whichever of accuracy or RT was available was used. If both were available, then the average of the two effect sizes (one for accuracy, one for RT) was used.

Tasks that require inhibiting a dominant response, such as go/no-go (Gomez et al., 2007; Wright et al., 2014) and were also eligible for Category 3. For go/no-go tasks accuracy only was eligible because this provides direct insight into the success of inhibiting the response. Commission errors were preferred, but overall accuracy was also eligible. Continuous performance tasks were considered equivalent to go/no-go tasks only if they included the requirement to withhold a response to certain stimuli (Wright et al., 2014). Here, again, the focus was on accuracy. Stop-signal tasks were also eligible, in which case stop-signal RT was used (Verbruggen & Logan, 2008).

Finally, Category 4 had only a single eligible task: The SART (Robertson et al., 1997; Vallesi et al., 2021). Here, the preferred outcome was commission errors, but if only an overall sensitivity or accuracy measure was available, then this was used. It is possible that the SART belongs in Category 3 along with other response inhibition tasks. However, given the intrinsic conceptual relationship between SART and CFQ, we initially assessed it as a separate category, and assessed during data-analysis whether it could be productively combined with Category 3.

All performance measures were coded such that higher scores reflected better performance on the task. Given that higher CFQ scores reflect greater cognitive failures (i.e., poorer cognitive function), this means that a negative correlation was expected between CFQ scores and performance measures. A bivariate correlation was the preferred effect size, but if only regression coefficients or partial correlations were available, then these were used, and +.05 was added to positive coefficients and −.05 was subtracted from negative coefficients to correct for the reduction in coefficient magnitude relative to bivariate correlations (Vaerenbergh, 2018). Where an *F*-value or *t-*value for higher and lower CFQ groups was all that was available, this *F*-value was converted to a Cohen’s *d* effect size using an online calculator (Lenhard & Lenhard, 2022), and then converted to a correlation coefficient (Lin, n.d.). All standard errors were calculated using 95% confidence intervals using an online tool provided by Psychometrica (Lenhard & Lenhard, 2014). As stipulated in Berkhout et al. (2024), effect sizes and standard errors were transformed to Fisher’s *z* to correct for the bounded nature of correlation coefficients.

## Results

### Eligible effect sizes and participant characteristics

A total of 88 works, spanning publication years from 1987 to 2024, contributed a total of 151 effect sizes met the above eligibility criteria and were included in the meta-analysis.

In Category 1, there were 37 effect sizes from 37 studies (Adjorlolo, 2016; Amidi et al., 2015; Baker et al., 2018; Bassel et al., 2002; Bellens et al., 2022; Berende et al., 2019; Costa et al., 2023; Das-Smaal et al., 1993; Dawson et al., 2014; Dey et al., 2019; Finn & McDonald, 2015; Friedman & Miyake, 2004; Gajewski & Falkenstein, 2012, 2014, 2015a; Gajewski et al., 2011, 2022; Gajewski, Thönes, et al., 2020b; Goodman et al., 2022; Guariglia et al., 2020; Gustavson et al., 2015; Hagen et al., 2020; Jozwiak et al., 2017; Keilp et al., 2013; Koso et al., 2012; Kramer et al., 1994; Kreitz et al., 2015; Lange & Süß, 2014; Lapadula et al., 2020; Miskowiak et al., 2021, 2022, 2023; Shakeel & Goghari, 2017; Slana Ozimič & Repovš, 2020; Steinbusch et al., 2017; Vom Hofe et al., 1998; Woods et al., 2024).^2^^,^^3^*N* = 6,092 participants contributed data to this category, including students, general community adults, healthy older adults (50+), older adults with mild or subjective cognitive impairment, older adults with vascular risk factors, older adults with amnesic mild cognitive impairment, twins, people previously hospitalised due to coronavirus disease (COVID-19) infection, cardiac arrest survivors, breast cancer survivors with cognitive complaints, combat veterans with posttraumatic stress disorder (PTSD), and people with: chronic back pain, long COVID, persistent symptoms attributed to Lyme disease, testicular cancer, Major Depressive Disorder (MDD), traumatic brain injury (TBI), and Parkinson’s disease. For this and all subsequent categories, the list of the specific task measures derived from each study and the participant type for each are provided in the Supplementary Material.

In Category 2, there were 46 references (Ahmad et al., 2022; Amidi et al., 2015; Bellens et al., 2022; Bertens et al., 2016; Bloomfield et al., 2010; Chang et al., 2021; Dawson et al., 2014; Dean & Sterr, 2013; Donohoe et al., 2009; Friedman & Miyake, 2004; Gajewski & Falkenstein, 2012, 2014, 2015a; Gajewski et al., 2011, 2022; Gajewski, Thönes, et al., 2020b; Gokal et al., 2018; Guariglia et al., 2020; Gustavson et al., 2015; Guye et al., 2020; Hagen et al., 2020; Hitchcott et al., 2017; Jensen et al., 2022; Jozwiak et al., 2017; Judah et al., 2014; Kim et al., 2014; Kitahata et al., 2017; Könen & Karbach, 2020; Krabbe et al., 2017; Kreitz et al., 2015; Lange & Süß, 2014; McVay & Kane, 2009; Miskowiak et al., 2022; Molteni et al., 2008; Montgomery & Fisk, 2007; Peers et al., 2022; Pollina et al., 1992; Richter et al., 2015; Rodriguez et al., 2013; Rossiter et al., 2006; Ryskin et al., 2015; Shakeel & Goghari, 2017; Torenvliet et al., 2022; Unsworth et al., 2012; Welhaf & Kane, 2024; Woods et al., 2024).^4^ These yielded 52 effect sizes, because five references had two separate effect sizes for different groups (e.g., clinical vs. control; Ahmad et al., 2022; Dean & Sterr, 2013; Krabbe et al., 2017; Rossiter et al., 2006; Torenvliet et al., 2022), and one reference reported two separate studies that were both eligible (Kreitz et al., 2015). *N* = 6,690 individuals contributed data to this category, including students, students with attention-deficit/hyperactivity disorder (ADHD), general community adults, healthy older adults (50+), older adults with mild or subjective cognitive impairment, twins, breast cancer survivors with cognitive complaints, stroke survivors, people who use Methylenedioxymethamphetamine (MDMA), and people with: work-related stress, stress-related exhaustion, long COVID, tinnitus, testicular cancer, breast cancer, MDD, autism, schizophrenia, TBI, vestibular loss, and Parkinson’s disease.

Category 3 included 41 references (Adjorlolo, 2016; Baker et al., 2018; Bellens et al., 2022; Berende et al., 2019; Costa et al., 2023; Dey et al., 2019; Forster & Lavie, 2007; Friedman & Miyake, 2004; Gajewski & Falkenstein, 2012, 2014, 2015a; Gajewski et al., 2011, 2022; Gajewski, Thönes, et al., 2020b; Gokal et al., 2018; Gunduz et al., 2022; Gustavson et al., 2015; Hagen et al., 2020; Horvat & Tement, 2020; Hsieh et al., 2022; Ishigami & Klein, 2009; Jozwiak et al., 2017; Judah et al., 2014; Kalpidou et al., 2021; Keilp et al., 2013; Könen & Karbach, 2020; Krabbe et al., 2017; Kramer et al., 1994; Kreitz et al., 2015; Lopresti et al., 2022, 2023; Polsinelli et al., 2020; Ryskin et al., 2015; Slagboom et al., 2021; Slana Ozimič & Repovš, 2020; Swick & Ashley, 2017; Tipper & Baylis, 1987; Vom Hofe et al., 1998; Welhaf & Kane, 2024; Woltering et al., 2013; Woods et al., 2024). These yielded 44 effect sizes because two references had two separate eligible studies (Ishigami & Klein, 2009; Kreitz et al., 2015), and one reference had two discrete groups of participants (Krabbe et al., 2017). *N* = 6,058 contributed data to this category, including students, students with low- and high-trait anxiety, students with ADHD, general community adults, healthy older adults (50+), people 40–75 with self-reported subjective cognitive issues, older adults with mild or subjective cognitive impairment, older adults with vascular risk factors, twins, breast cancer survivors with cognitive complaints, combat veterans with PTSD, and people with: chronic back pain, stress-related exhaustion, persistent symptoms attributed to Lyme disease, hypopituitarism, breast cancer, MDD, TBI, and Parkinson’s disease.

In Category 4, sixteen references (Bloomfield et al., 2010; Dockree et al., 2006; Donohoe et al., 2009; Farrin et al., 2003; Gokal et al., 2018; Koso et al., 2012; Larue et al., 2010; Linden et al., 2005; Manly et al., 1999; McVay & Kane, 2009; Righi et al., 2009; Robertson et al., 1997; Smilek et al., 2010; Wallace et al., 2001; Whyte et al., 2006; Zanesco et al., 2019) contributed 18 effect sizes, because two references had two discrete groups (Dockree et al., 2006; Whyte et al., 2006). *N* = 1,448 individuals contributed data to this category, including students, general community adults, military personnel, combat veterans with PTSD, and people with: work burnout, breast cancer, schizophrenia, and TBI.

### Bayesian meta-analysis: All eligible effects

Values were rounded to two decimal places for calculations. Analysis was performed in JASP (Version 0.14.1). We performed four random-effects Bayesian meta-analyses with default priors, one for each of the four categories of executive function tasks. Bayes factors reflected the evidence for the alternative hypothesis (that CFQ scores and performance were associated, no direction assumed) divided by the evidence for the null hypothesis (that CFQ scores and performance were not related). Therefore, values greater than 1 reflect greater evidence for the alternative hypothesis while values less than 1 reflect greater evidence for the null hypothesis. The strength of evidence provided by different Bayes factors were interpreted consistent with the recommendations in van Doorn et al. (2021). That is, a BF_10_ greater than 3 reflects moderate evidence for alternative hypothesis (i.e., 3× evidence for alternative vs. null), while a BF_10_ less than 1/3 reflects moderate evidence for the null (i.e., 3× more evidence for null vs alternative). Values less than 3 and greater than .33 reflect an indeterminant outcome, or weak evidence for the alternative and null hypotheses respectively. BF_10_ > 10 and < .1 reflect strong evidence for the alternative and null hypotheses, respectively.

The first analysis included variants of the CFQ and all types of effect size. The results of this first analysis are provided in Table 2. In subsequent sensitivity analyses, we assessed the influence of removing outliers, restricting only to studies that used the original 25-item CFQ (Broadbent et al., 1982), and restricting only to studies that provided bivariate correlations. Note for all studies included in the meta-analyses, information about the individual studies including the specific measures they used are provided in theSupplementary Material.

**Table 2:** Results of Bayesian meta-analysis for each of the four categories of executive function tasks with any CFQ

Category	μ	BF_10_	τ	BF_10_
Switching	−.11 [−.19, −.03]	1.66	.22 [.17, .30]	5.19e^48^
Updating WM	−.07 [−.10, −.04]	30.87	.07 [.03, .11]	.70
Inhibition	−.08 [−.14, −.03]	1.88	.16 [.12, .22]	6.04e^9^
SART	−.19 [−.28, −.09]	18.03	.15 [.07, .26]	72.44

*Note.* Square brackets provide 95% credible interval. WM = working memory; SART = Sustained Attention to Response Task; μ = mean; τ = standard deviation; BF_10_ = random effects H_1_/random effects H_0_

Table 2 shows that all four categories produced negative mean effects (i.e., correlations), consistent with individuals more prone to cognitive failures demonstrating worse objective task performance. The BF_10_ values indicated that there was only weak evidence for the effects for switching and inhibition. In contrast, there was strong support for the effects for Updating Working Memory and SART.

### Bayesian meta-analysis: Outliers excluded

Despite the diversity of eligible tasks for the updating working memory category, there was minimal variance in the effect sizes in this category. However, given the large heterogeneity present in many of the categories, boxplots of effect sizes were used to identify outliers within each category. Based on this, *N* = 3 effect sizes were excluded from Category 1, *N* = 3 from Category 2, *N* = 3 from Category 3, and none from Category 4. It appeared that most if not all these effect size outliers were derived from studies with lower sample sizes, and thus a sampling error likely contributed to them being outlier values. That is, for Category 1, these outlier effect sizes all arose from sample sizes smaller than the average for the category (*N* = 36, *N* = 12, and *N* = 70; Bassel et al., 2002; Miskowiak et al., 2022; Vom Hofe et al., 1998), where the mean was *N =* 165, median = 91. Similarly, for Category 2, these outlier effect sizes also all arose from sample sizes smaller than the mean for the category (*N* = 14, *N* = 25, and *N* = 37, where mean *N* = 129, median = 75; Ahmad et al., 2022; Chang et al., 2021). Further, for Category 3, these outlier effect sizes were also all derived from sample sizes smaller than average for the category (*N* = 62, *N* = 25, and *N* = 32, where mean *N* = 138, median *N* = 69; Costa et al., 2023; Krabbe et al., 2017; Tipper & Baylis, 1987). The study with *N* = 62 in Category 3 also derived from (a) a factor of the CFQ rather than the full original CFQ, (b) from regression weights rather than bivariate correlations, and (c) Stroop incongruent trials rather than interference scores (Costa et al., 2023). These factors may have also contributed to making that effect size an outlier.

The results of the meta-analyses following these exclusions are provided in Table 3. The forest plots are large and thus are provided in the Supplementary Material. Table 3 shows that once outliers were excluded, now inhibition also showed strong evidence for a mean effect, along with updating working memory and SART. Switching now showed weak evidence in favour of the null hypothesis of no relationship. There appeared to be substantive reduction in heterogeneity in some categories once these small number of outliers were excluded (e.g., now confidence intervals around τ for both switching and inhibition did not overlap their confidence intervals from previous analysis). Further, as discussed above, these outlier effect sizes appeared to be products of studies with smaller sample sizes producing unreliable estimates of effect size. Therefore, this analysis with the outliers excluded was considered the primary results, and all subsequent analyses were performed with these outliers excluded to provide representative and robust estimates of effect sizes.

**Table 3:** Results of Bayesian meta-analysis for each of the four categories of executive function tasks with any CFQ after outlier exclusion

Category	μ	BF_10_	τ	BF_10_
Switching	−.06 [−.11, −.01]	.50	.11 [.07, .16]	26,893.21
Updating WM	−.06 [−.10, −.03]	17.80	.05 [.03, .09]	.16
Inhibition	−.07 [−.11, −.03]	15.40	.07 [.03, .11]	1.68
SART	−.19 [−.28, −.09]	18.03	.15 [.07, .26]	72.44

*Note.* Square brackets provide 95% credible interval. WM = working memory; SART = Sustained Attention to Response Task; μ = mean; τ = standard deviation; BF_10_ = random effects H_1_/random effects H_0_

It could be argued that SART contains elements of response inhibition, akin to that captured by tasks such as go/no-go in Category 3. To test this, we examined the effect of combining Category 3 and Category 4. While the mean effect was clear (μ = −.11, [−.15, −.07], BF_10_ = 3,321.19), the combined τ was halfway between the two individual τs suggesting that there was no synergistic benefit to combining them, the confidence intervals for τ still overlapped that of both original estimates, suggesting that there was no clear reduction in heterogeneity from combining them, and there was greater evidence for heterogeneity than for either category in isolation (τ = .11, [.08, .15], BF_10_ = 6.334e^6^). Therefore, they were treated as separate categories for the remaining analyses.

To test for potential publication bias, funnel plots were generated via classical meta-analysis with restricted ML method examined, and rank correlation test for plot asymmetry was performed. None of the plots suggested publication bias, and none of the rank tests were significant (*p*-values ≥ .176), indicating no evidence of publication bias, except for inhibition, which was significant (*p* = .047). This was such that negative correlations may have been overrepresented. However, additional analyses on subsets of studies below eliminated evidence for publication bias in this category, but there was still clear evidence for the effect size for this category. Thus, the presence of a reliable effect is not attributable to publication bias.

### Bayesian meta-analysis: Outliers excluded and restricted to original CFQ

In the previous analyses, variants of the CFQ were eligible for inclusion. While most studies used the original CFQ, some did not. Specifically, in switching, four out of 31 used a variant of the CFQ, in updating working memory, eight out of 49 used variants, in inhibition, five out of 41 used variants, and in SART, three out of 18 used a variant. Given the relatively small number of studies using variants, to assess whether CFQ type affected the results, the next analysis was focussed exclusively on studies that used the original CFQ only (not variants of the CFQ). The results for all categories are shown in Table 4.

**Table 4:** Results of Bayesian meta-analysis for each of the four categories of executive function tasks with original CFQ only

Category	μ	BF_10_	τ	BF_10_
Switching	−.07 [−.13, −.02]	1.25	.10 [.06, .16]	5,698.97
Updating WM	−.06 [−.10, −.03]	3.50	.06 [.03, .10]	.23
Inhibition	−.08 [−.13, −.04]	24.40	.08 [.04, .13]	3.49
SART	−.20 [−.32, −.08]	6.39	.18 [.08, .31]	90.10

*Note.* Square brackets provide 95% credible interval. WM = working memory; SART = Sustained Attention to Response Task; μ = mean; τ = standard deviation; BF_10_ = random effects H_1_/random effects H_0_

Here, there was strong evidence for the mean effect for inhibition and moderate evidence for the presence of an effect for updating working memory and SART. There was still only weak support for switching. Further, in this analysis, there was no longer evidence for publication bias in Category 3 (inhibition) (*p* = .139).

### Bayesian meta-analysis: Outliers excluded and restricted to bivariate correlations only

The most accurate effect size derivation is from bivariate correlations. Regression coefficients require an adjustment for the other variables in the analysis, and this adjustment is likely going to be an imperfect correction when the true bivariate relationship is not known. Therefore, to check that such corrections had not distorted the outcome, next we performed an additional analysis deriving effect sizes only from bivariate correlations, not partial correlations, regression coefficients, or group-difference statistics. We returned to considering all CFQ types for these analyses. The results are shown in Table 5.

**Table 5:** Results of Bayesian meta-analysis for each of the four categories of executive function tasks with any CFQ and only bivariate correlation effects

Category	μ	BF_10_	τ	BF_10_
Switching	−.07 [−.12, −.02]	.90	.10 [.06, .16]	19,406.53
Updating WM	−.07 [−.10, −.04]	57.24	.05 [.02, .08]	.09
Inhibition	−.07 [−.11, −.03]	15.40	.07 [.03, .11]	1.68
SART	−.19 [−.29, −.07]	5.75	.16 [.07, .27]	40.35

*Note.* Square brackets provide 95% credible interval. WM = working memory; SART = Sustained Attention to Response Task; μ = mean; τ = standard deviation; BF_10_ = random effects H_1_/random effects H_0_

Here, there was moderate evidence for the presence of an effect for SART, and strong evidence for updating working memory and inhibition. There was weak evidence in favour of the null hypothesis for switching.

### Bayesian meta-analysis: Reducing heterogeneity for switching

Finally, we assessed whether the heterogeneity in switching could be reduced by focusing only on studies that used the same outcome measure. The Trail Making Test B was the most commonly used metric in this category, and therefore we focussed the analysis exclusively on this category. In addition, to promote homogeneity, the analysis was focused only on original CFQ items and only bivariate correlations. This left *N* = 19 studies for analysis. Although there was minimal change in τ value itself, the strength of evidence for heterogeneity was now substantively reduced, (τ = .12, [.06, .20], BF_10_ = 49.77), but there was still only weak evidence for the switching effect size (μ = −.10, [−.17, −.03], BF_10_ = 1.85). This means that although we reduced the magnitude of evidence for it (from BF_10_ = 19,406.53 to BF_10_ = 49.77), we were not able to eliminate the strong evidence for heterogeneity within the switching condition. The SART was another category which consistently showed strong evidence for heterogeneity across analyses. This suggests that there are likely additional variables that moderate the strength of the observed relationship in these categories. As discussed below, measurement reliability is one such candidate.

## Results summary

To summarise, in every analysis, there was evidence that multiple aspects of objective performance on executive function were related to CFQ scores. These effects were such that individuals more prone to subjective cognitive failures (i.e., higher CFQ scores) were associated with poorer objective performance on these executive function tasks. With respect to the specific categories, there was not consistent evidence that switching was related to CFQ scores. Instead, the evidence for this category percolated around an indeterminant zone of weak evidence for either the null or alternative hypothesis. There was consistently moderate or strong evidence for updating working memory and SART being related to CFQ scores. Once outliers were removed, there was also consistently strong evidence for inhibition. There was some potential evidence for publication bias for inhibition, but even in analyses where this evidence was eliminated (i.e., focus on original CFQ only), there was still clear evidence for a robust effect size for inhibition.

## Discussion

Here, we used Bayesian meta-analyses to assess the relationship between scores on the CFQ and objective performance on executive function tasks that fell into one of four categories. Three categories were derived from an influential taxonomy of executive function (i.e., switching, updating working memory, and inhibition; Miyake et al., 2000), and an additional one specifically for SART was included because this task was purpose-made to detect cognitive failures (Robertson et al., 1997). We assessed the robustness of effects across multiple analytic choices. There was consistently moderate or strong evidence for CFQ scores being related to performance on updating working memory tasks and SART, and once outliers were removed, there was also strong evidence for tasks gauging inhibition. In contrast, there was never definitive evidence for the relationship between CFQ score and performance on tasks that are designed to measure switching.

These results indicate that people do have a degree of insight into their cognitive function, and how they subjectively experience it playing out in everyday life is systematically related to how it influences objective performance on tasks designed to measure some aspects of executive function. However, an important qualification is that the category of task matters: This relationship was only robust for certain types of tasks and not others. Consistent with SART being developed to detect the absent-minded lapses in attention that are reflected in the content of the CFQ, there was strong evidence for a CFQ–SART relationships. Indeed, numerically at least, SART consistently produced the strongest relationships. This is consistent with recent evidence that the CFQ shows reliable associations with low-prevalence visual search (Goodhew & Edwards, 2023; Thomson & Goodhew, 2021)—a special kind of visual search tasks where targets occur infrequently and which results in an elevated target miss rate (Horowitz, 2017; Wolfe et al., 2005). While such studies are not pure SART tasks and thus were not included in the present meta-analysis, the fact that both SART and low-prevalence search performance correlates with CFQ suggests that there may be some shared variance between SART and low-prevalence visual search. We suggest that this is likely because they both develop participants’ expectations about a particular type of outcome being the most common, and then infrequently require deviating from this expectation, and it is this flexibility that individuals prone to cognitive failures appear to have difficulty with. This is consistent with some CFQ items reflecting inflexibility in adapting to changing context, such as failing to hear people speaking to you when doing something else. Here, the person has focussed their attention in one way, and is inefficient and/or ineffective in changing this focus as required.

Further, the results indicated that individuals prone to cognitive failures have difficulty juggling the demands of complex span tasks including updating and performing operations on the contents of their working memory. Working memory facilitates effective and strategic regulation of attention in the pursuit of goals (e.g., Bleckley et al., 2003; Burgoyne et al., 2023; Robison & Unsworth, 2017). In this light, it is interesting that multiple CFQ items refer to issues with goal pursuit, including a failure to initiate goals (e.g., leaving important letters unanswered for days), failure to properly execute a goal (e.g., throwing away the thing you meant to keep and keeping the thing you meant to throw away), and losing track of goals as the context changes (e.g., forgetting why you went from one part of the house to another, and forgetting what you came to the shops to buy). Given the function of working memory, in conjunction with the negative relationship between updating working memory and CFQ scores observed in the meta-analysis, it is likely that poorer ability to update working memory is implicated in these issues with initiating, maintaining, and successfully executing goals that are apparent in those prone to cognitive failures.

In a similar vein, the present findings indicate that individuals prone to cognitive failures also have difficulty inhibiting salient stimuli or inhibiting a response. This is consistent with CFQ items reflecting failures to apply attention to important sources of information (e.g., failing to notice signposts on the road, daydreaming when you ought to be listening to something, and failing to see what you want in the supermarket even though it is there), and failing to inhibit responses (e.g., losing one’s temper and regretting it). This suggests that inhibition is an important aspect of a person’s overall cognitive function in everyday life.

What individuals prone to cognitive failures did *not* appear to have clear difficulty doing is *switching* tasks or attentional sets. Across our analyses, the Bayes factors never deviated from the weak/indeterminant zone of evidence to provide definitive support for the presence (or absence) of this relationship. This seems surprising, given that CFQ scores were related to all other aspects of executive function. It is possible that the CFQ is truly unrelated to switching. None of the items refer explicitly to difficulty starting a new task after doing a different one. Although some refer to the opposite—accidentally switching tasks, such as *Do you start doing one thing at home and get distracted into doing something else (unintentionally)?—*this is different from a carryover where a previous task negatively impacts a new one. Alternatively, it is possible that CFQ scores are related to switching performance but that the methodology of the studies in the meta-analysis obscured this relationship. One possible methodological explanation for this is that since most effect sizes in this category were derived from the Trail Making Test B, this measure may be not optimally sensitive to individual differences in switching. Indeed, the Trail Making Test B derives from the neuropsychological literature, and therefore it may be better suited to detecting gross cognitive impairment following brain injury rather than making subtle differentiation between task-switching ability. It may be that CFQ scores would be robustly related to switching task performance if more sensitive measurements of switching were used that were better able to finely differentiate between individuals’ switching capacities. All we can say at this point is that there is not definitive evidence that CFQ scores are related (or not related) to switching performance.

While here we focussed on the dissociable components of executive function (switching, updating, and inhibition), there is also evidence of a common shared variance between all executive functions (e.g., Miyake & Friedman, 2012). The shared (unity) versus dissociable (diversity) variance of executive functions can be gauged via latent variable modelling. This approach addresses the ‘task impurity’ problem, which is that while some of the variance in performance on a specific executive function task reflects a person’s executive function, task-specific factors that do not gauge executive function also contribute variance to performance (e.g., colour-naming ability in Stroop). Having participants complete multiple measures of each of the dissociable components of executive function allows the shared versus dissociable components of executive function to be distinguished from one another and from task-specific variance via latent variable modelling (Miyake & Friedman, 2012; Miyake et al., 2000). Unfortunately, this was not feasible to do for this meta-analysis, given that most studies did not have the requisite measures and/or individual-level data available. However, we think this would be a valuable future research direction to further elucidate how the CFQ relates to performance on executive function tasks. In particular, for switching, it would be informative to determine whether it is related to CFQ scores when multiple (potentially more sensitive) measures of switching are used, and switching is quantified via latent variable modelling. If multiple measures of other aspects of executive function were also included, then it would also inform whether CFQ is most strongly related to general executive function rather than the dissociable components, which is quite possible given the breadth of cognitive domains captured by the CFQ. In this light, it is interesting that higher CFQ scores are associated with difficulties in emotion regulation (Robins et al., 2012) including reduced use of reappraisal emotion regulation strategy (Goodhew & Edwards, 2024b), and reappraisal ability has been shown to have selective relationships with general executive function (Toh & Yang, 2022).

It is also important to acknowledge that none of the effect size estimates here were large. In the following sections, we discuss some methodological as well as theoretical reasons that could explain this. When there is divergence between subjective measures and measures derived from objective performance on experimental tasks, subjective measures are often blamed. But it is important to consider reasons that objective measures may contribute to such divergence. Here, we will consider four such reasons. The first is measurement reliability. The CFQ typically shows good measurement reliability. In contrast, many experimental tasks designed to assess executive function—especially those that were first designed to show large-magnitude effects in the whole sample—can have poor measurement reliability when it comes to consistently differentiating between individuals (Friedman & Gustavson, 2022; Goodhew & Edwards, 2019; Hedge et al., 2018). This is because an effect that is experienced strongly by most or all in the sample typically has low between-participant variability, and between-participant variability is a prerequisite for individual differences reliability. Indeed, tasks such as the flanker task and Stroop task, both of which were eligible for the inhibition category, have been critiqued for having poor measurement reliability (Hedge et al., 2018).^5^ This is important because the measurement reliability of both the subjective and experimental measure will ultimately constrain the maximum correlation that can be observed between them (e.g., Dang et al., 2020; Parsons et al., 2019; Spearman, 1910), and therefore poor measurement reliability of experimental measures may have led to an underestimation of the magnitude of the association between CFQ scores and objective performance on at least some of the task categories here. It is so rare for the measurement reliability of experimental tasks to be explicitly reported that we could not perform any systematic correction for measurement reliability here, and thus it remains a viable cause of effect size underestimation. In this light, it is impressive that despite these issues, we still observed robust relationships between CFQ and objective performance on multiple aspects of executive function.

Second, subjective measures also typically aggregate information over a far larger times cale (e.g., CFQ asks about experiences in the previous 6 months), whereas objective performance on executive function tasks is usually assessed at a single point in time (Duckworth & Kern, 2011). This means that not only is the experimental measure more limited in its scope but it is also potentially more affected by state factors (e.g., sleep deprivation the previous night) that muddy estimates of a person’s trait executive function ability. These factors may contribute to the poor measurement reliability of some experimental measures, thus limiting the maximum observable correlation.

Third, subjective measures often capture a broader range of cognitive function, in contrast to experimental tasks, which typically reflect just a narrow snapshot of cognitive function, often with impoverished or artificial stimuli (Carrigan & Barkus, 2016; Dang et al., 2020; Toplak et al., 2013). For example, the CFQ asks about failures across a diverse array of aspects of executive function (e.g., prioritising the wrong information stream, succumbing to distracting information, failing to initiate goals, failing to maintain goals across contexts), whereas most experimental executive function tasks operationalise just a single aspect (e.g., Stroop task assess succumbing to distracting information). Further, while the CFQ collates information across a variety of contexts (e.g., driving, social settings, shopping), behavioural measures are derived from an experimental context with impoverished stimuli in artificial set-ups which often do not resemble the stimuli or tasks of everyday life (e.g., most adults do not spend significant time naming colours). These differences mean that we would not necessarily expect a high degree of convergence between the subjective and experimental measures, and the subjective measures may provide the more meaningful insight.

Fourth, while performance-based measures likely gauge *optimal* or *maximal* function where the experimental context provides clear structure and requirements, subjective measures poll information about *typical* everyday usage in less structured contexts (Dang et al., 2020; Friedman & Gustavson, 2022; Toplak et al., 2013). It is possible that executive function deficits may be more pronounced in typical usage in the relatively unstructured contexts of everyday life. In this vein, it is interesting to note that subjective cognitive decline in older adults appears to precede and predict objectively measurable decline in cognitive performance in the years ahead (Mitchell et al., 2014).

Of course, however, subjective measures are vulnerable to several key sources of measurement error, including self-presentation biases and failures of metacognitive insight. There are some situations in which these sources of error will fatally undermine the validity of a subjective measure (e.g., contexts where there is overwhelming motivation to present as capable, or where metacognitive insight is severely impaired; Friedman & Gustavson, 2022). However, one key advantage of the CFQ over other measures of subjective cognitive function, such as the Attentional Control Scale (ACS; Clarke & Todd, 2021; Derryberry & Reed, 2002; Judah et al., 2014), is that the item design minimises the role of metacognitive insight by focusing on the *outcomes* of cognitive function, which may improve its validity (Goodhew & Edwards, 2024b; Thomson & Goodhew, 2021). That is, the ACS requires participants to engage in sophisticated metacognition and comparison with others to answer questions such as “it is easy for me to alternate between two tasks.” There is clear evidence that humans experience a task-switching cost (e.g., Monsell, 2003), so an individual with astutely tuned metacognitive insight would appreciate this cost and thus may not endorse a statement about finding it easy to switch between tasks even though they are relatively good at it. To endorse this item, they need to know how their task-switching cost (or subjective feelings in relation to task switching) compares with that of other individuals, and such information is likely difficult to ascertain. If they do not endorse such items, then their ACS score would underestimate their true attentional control ability. In contrast, the CFQ simply requires participants to remember instances of failures of attentional control, such as bumping into people and forgetting appointments. Of course, a person with severe memory impairment may forget instances of forgetting. But provided that overall memory is not severely degraded, CFQ items are likely less demanding of metacognitive insight than other subjective cognition measures like the ACS. Reducing the contribution of metacognitive insight to scores on a subjective cognitive measure will (all other things being equal) reduce error variance and thus increase their validity. In this light, it is interesting that CFQ scores have been shown to predict low prevalence visual search performance when ACS do not (Thomson & Goodhew, 2021).

Ultimately, subjective and objective performance measures may gauge different aspects of cognitive function, and therefore, the greatest utility may be obtained by considering the information offered by both types of measures, rather than relying on either in isolation (e.g., Duckworth & Seligman, 2005; Friedman et al., 2020; Toplak et al., 2013). It may be particularly revealing to consider when and how they converge versus diverge. However, if a researcher faces time and resource constraints, then in many instances subjective measures are going to provide a more reliable and practical-to-obtain estimate (Duckworth & Kern, 2011). The CFQ in particular has a lot to offer.

However, the CFQ is many decades old, and many of the items no longer have the same contemporary relevance (e.g., referring to physical newspapers), and it lacks items that capture many modern instances of cognitive failures (e.g., forgetting passwords). We recently developed a contemporary measure of cognitive failures—the CFQ 2.0 (Goodhew & Edwards, 2024a). It consists of 15 items that are a mix of original CFQ items and new contemporary items. We found that the CFQ 2.0 outperformed the original CFQ in explaining variance in objective performance in low-prevalence visual search (Goodhew & Edwards, 2024a). Researchers should consider using this updated CFQ so that the CFQ can continue to maintain and even enhance its validity.

In conclusion, we performed Bayesian meta-analyses on the relationship between CFQ scores and objective performance on executive function tasks in four categories—switching, updating working memory, inhibition, and SART. There was robust evidence for the relationship between CFQ scores and performance on SART, updating working memory, and inhibition tasks, whereas there was not definitive evidence either for or against the relationship between CFQ and performance on switching tasks. This could be because the primary method of measuring switching in the studies included was the Trail Making Test B, which may be more suited to detecting gross dysfunction than subtle individual differences in switching ability. Altogether, this suggests that subjective cognitive function is associated with objective performance on executive function tasks, but it is important to consider the type of cognitive task when examining this association. It is also important to consider the different information about cognitive function provided by subjective measures like the CFQ compared with measures derived from performance on experimental tasks. At least in some cases, the CFQ can offer richer and more reliable insight into cognitive function.

## Supplementary Information

Below is the link to the electronic supplementary material.

Supplementary file1 (DOCX 1236 KB)

## Data Availability

Some additional data are provided in the Supplementary Material. The full raw dataset is available upon request.

## Footnotes

1 This is also known as “shifting” (the two terms are used interchangeably in the literature). Here, we prefer the term “switching” because this maps onto task-switching, which is one of the core aspects of this aspect of executive function (at least to us, task-*shifting* sounds peculiar).

2 For Jozwiak et al. (2017) for this and subsequent categories, correlation was based on a subset of original data that was available for reanalysis.

3 For Gajewski and Falkenstein (2015a) that was identified in original search, when providing the correlations an author clarified that same sample is used in Gajewski and Falkenstein (2015b) and correlations reported here are from both papers. For simplicity, we count this as a single reference and cite Gajewski and Falkenstein (2015a) throughout.

4 For Kim et al. (2014), an author clarified that they were providing the correlation based on a series of studies in the laboratory all with Digit Span backwards and on students with ADHD, including but not limited to Kim et al. (2014). For simplicity, we count this as a single reference, since there was a single effect size resulting.

5 Hedge et al. (2018) attributed the poor measurement reliability to low between-participant variability. In contrast, Gajewski, Getzmann et al. (2020a), Gajewski, Thönes et al. (2020b) observed large between-participant variability in Stroop interference across different age groups, although they did not report estimates of measurement reliability. More broadly, the measurement reliability of any measure depends on an interaction between specific contextual variables (e.g., number of trials used) and the characteristics (e.g., traits) of the people completing the task, and thus there is not a single measurement reliability value for the Stroop task. However, there is clear evidence that at least in some instances, its measurement reliability can be low, and there is reason to think that this is true for many of studies considered here. Interested readers are referred to Burgoyne et al. (2023) for a modified Stroop task that is highly reliable.
