# Supplementary material for: A meta-analysis on the relationship between subjective cognitive failures as measured by the cognitive failures questionnaire (CFQ) and objective performance on executive function tasks
Source: Psychon Bull Rev. 2024 Sep 9;32(2):528–46. doi: 10.3758/s13423-024-02573-6 (PMC12000218; doi:10.3758/s13423-024-02573-6)

**Supplementary Material**

***Category 1 (Switching):***

| **Reference** | **Effect Size Type** | **Participant Type** | **CFQ Type** | **Measure(s) included** |
| --- | --- | --- | --- | --- |
| Adjorlolo et al. (2016) | 1 | 18 | 1 | TMT-B |
| Amidi et al. (2015) | 1 | 31 | 1 | TMT-B, WCST (PE) |
| Baker et al. (2018) | 1 | 16 | 1 | TMT-B and WCST (PE) |
| Bassel et al. (2002) | 1 | 15 | 1 | TMT-B |
| Bellens et al. (2022) | 1 | 7 | 1 | TMT-B |
| Berende et al. (2019) | 1 | 32 | 6 | TMT-B |
| Costa et al. (2023) | 2 | 2 | 2 | WCST (PE) |
| Das-Smaal et al. (1993) | 1 | 1 | 1 | Star Counting Task (had to switch between backward and forwards counting) |
| Dawson et al. (2014) | 1 | 10 | 1 | WCST (PE) |
| Dey et al. (2019) | 1 | 35 | 1 | TMT-B |
| Finn & McDonald (2015) | 1 | 22 | 1 | TMT-B |
| Friedman & Miyake (2004) | 1 | 1 | 1 | Category Switch Cost, Global-Local Switch Cost, Number-Letter Switch Cost |
| Gajewski & Falkenstein (2012) | 1 | 2 | 1 | TMT-B |
| Gajewski & Falkenstein (2014) | 1 | 26 | 1 | TMT-B |
| Gajewski & Falkenstein (2015) | 1 | 2 | 1 | TMT-B |
| Gajewski et al. (2011) | 1 | 2 | 1 | TMT-B |
| Gajewski et al. (2020) | 1 | 2 | 1 | TMT-B |
| Gajewski et al. (2022) | 1 | 6 | 1 | TMT-B |
| Goodman et al. (2022) | 1 | 6 | 1 | WCST, TMT-B, Task Switching |
| Guariglia et al. (2020) | 1 | 6 | 1 | Rules Shift Cards |
| Gustavson et al. (2015) | 1 | 30 | 1 | 3 Task Switching Tasks (switch cost) |
| Hagen et al. (2020) | 1 | 29 | 1 | TMT-4 |
| Jozwiak et al. (2017) | 1 | 38 | 1 | TMT-B |
| Keilp et al. (2013) | 1 | 33 | 1 | WCST (errors) |
| Koso et al. (2012) | 2 | 34 | 1 | TMT-B |
| Kramer et al. (1994) | 1 | 26 | 1 | WCST (PE) |
| Kreitz et al. (2015) - Study 2 | 1 | 1 | 4 | Navon Switch (%increase in RT Switch) |
| Lange & Süß (2014) | 1 | 2 | 4 | Task Switching (switch cost) |
| Lapadula et al. (2020) | 1 | 15 | 1 | TMT-B |
| Miskowiak et al. (2021) | 1 | 12 | 1 | TMT-B |
| Miskowiak et al. (2023) Brain Sciences | 1 | 5 | 1 | TMT-B |
| Miskowiak et al. (2023) Psychological Research | 1 | 5 | 1 | TMT-B |
| Shakeel et al. (2017) | 1 | 2 | 1 | TMT-B |
| Slana & Repovs (2020) | 1 | 6 | 1 | TMT-B |
| Steinbusch et al. (2017) | 1 | 19 | 1 | TMT-B |
| Vom Hofe et al. (1998) | 1 | 1 | 1 | TMT-B |
| Woods et al. (2024) | 1 | 6 | 1 | TMT-B |

*Note.* WCST = Wisconsin Card Sorting Test; PE = perseverative errors; TMT = Trail Making Test

*Note.* Nominal numeric codes are provided below following all category tables.

**Category 2 (Updating Working Memory)**

| **Reference** | **Effect Size Type** | **Participant Type** | **CFQ Type** | **Measure(s) included** |
| --- | --- | --- | --- | --- |
| Ahmad et al. (2022) bilateral vestibular loss patients | 1 | 3 | 1 | CBT-R |
| Ahmad et al. (2022) unilateral vestibular loss patients | 1 | 4 | 1 | CBT-R |
| Amidi et al. (2015) | 1 | 31 | 1 | Digit Span Backwards, PASAT |
| Bellens et al. (2022) | 1 | 7 | 1 | n-back |
| Bertens et al. (2016) | 1 | 18 | 1 | Letter Number Sequencing |
| Bloomfield et al. (2010) | 1 | 18 | 1 | PASAT and Letter Number Sequencing |
| Chang et al. (2021) | 1 | 10 | 1 | Digit Span Backwards |
| Dawson et al. (2014) | 1 | 10 | 1 | Digit Span Backwards |
| Dean & Sterr (2013) - controls | 1 | 6 | 1 | n-back and PVST |
| Dean & Sterr (2013) - TBI patients | 1 | 18 | 1 | n-back and PVST |
| Donohoe et al. (2009) | 1 | 23 | 1 | Letter Number Sequencing |
| Friedman & Miyake (2004) | 1 | 1 | 1 | Reading Span |
| Kim et al. (2014) & others | 1 | 20 | 1 | Digit Span Backwards |
| Gajewski & Falkenstein (2012) | 1 | 2 | 1 | Digit Span Backwards |
| Gajewski & Falkenstein (2014) | 1 | 26 | 1 | Digit Span Backwards |
| Gajewski & Falkenstein (2015) | 1 | 2 | 1 | Digit Span Backwards |
| Gajewski et al. (2011) | 1 | 2 | 1 | Digit Span Backwards |
| Gajewski et al. (2020) | 1 | 2 | 1 | Digit Span Backwards |
| Gajewski et al. (2022) | 1 | 6 | 1 | Digit Span Backwards and n-back (2-back d') |
| Gokal et al. (2018) | 1 | 17 | 1 | Digit Span Backwards |
| Guariglia et al. (2020) | 1 | 6 | 1 | Digit Span Backwards, CBT-R |
| Gustavson et al. (2015) | 1 | 30 | 1 | Spatial n-back |
| Guye et al. (2020) | 1 | 2 | 4 | Complex spatial span (like Symmetry Span) |
| Hagen et al. (2020) | 1 | 29 | 1 | Digit Span Backwards |
| Hitchcott et al. (2017) | 1 | 6 | 5 | Digit Span Backwards |
| Jensen et al. (2022) | 1 | 8 | 1 | Letter-Number Sequencing |
| Jozwiak et al. (2017) | 1 | 38 | 1 | Digit Span Backwards |
| Judah et al. (2014) - Study 3 | 1 | 1 | 1 | Letter-Number Sequencing |
| Kitahata et al. (2017) | 1 | 17 | 1 | Letter-Number Sequencing & Visual Memory (backwards) |
| Könen & Karbach (2020) | 1 | 6 | 1 | CBT-R, Digit Span Backwards |
| Krabbe et al. (2017) controls | 1 | 6 | 1 | n-back omissions and PVST |
| Krabbe et al. (2017) stress-related exhaustion patients | 1 | 37 | 1 | n-back omissions and PVST |
| Kreitz et al. (2015) - Study 1 | 1 | 1 | 4 | n-back (2 versions), AOSPAN |
| Kreitz et al. (2015) - Study 2 | 1 | 1 | 4 | n-back (2 versions), AOSPAN |
| Lange & Süß (2014) | 1 | 2 | 4 | aggregate of 3 complex span tasks (RSPAN, OSPAN, dot span) and updating tasks |
| McVay & Kane (2009) | 1 | 1 | 3 | OSPAN, SSPAN, RSPAN |
| Miskowiak et al. (2022/2023) Brain Sciences | 1 | 5 | 1 | Letter-Number Sequencing |
| Molteni et al. (2008) | 1 | 6 | 1 | n-back errors |
| Montogomery & Risk (2007) | 1 | 25 | 1 | Computation Span (variant of OSPAN) |
| Peers et al. (2022) | 1 | 28 | 1 | Spatial Span Reverse, Dot-matrix Reverse |
| Pollina et al. (1992) | 1 | 1 | 7 | Digit Span Backwards |
| Richter et al. (2015) | 1 | 18 | 1 | Digit Span Backwards, 2-back |
| Rodriguez et al. (2013) | 2 | 1 | 1 | AOSPAN, RSPAN |
| Rossiter et al. (2006) - control group | 1 | 6 | 1 | RSPAN |
| Rossiter et al. (2006) - tinnitus group | 1 | 36 | 1 | RSPAN |
| Ryskin et al. (2015) | 1 | 1 | 1 | OSPAN |
| Shakeel et al. (2017) | 1 | 2 | 1 | Digit Span Sequencing |
| Torenvliet et al. (2022) Autism group | 1 | 13 | 1 | n-back |
| Torenvliet et al. (2022) control group | 1 | 6 | 1 | n-back |
| Unsworth et al. (2012) | 1 | 1 | 1 | Composite of OSPAN, SSPAN, and RSPAN |
| Welhaf & Kane (2023/2024) | 1 | 1 | 3 | OSPAN, SSPAN, RSPAN |
| Woods et al. (2024) | 1 | 6 | 1 | Digit Span Backwards |

*Note.* CBT-R = Corsi Block Tapping (Reverse); PASAT = Paced Auditory Serial Addition Test; PVST = Paced Visual Serial Addition Task; OSPAN = Operation Span; RSPAN = Reading Span; SSPAN = Symmetry Span

**Category 3 (Inhibition)**

| **Reference** | **Effect Size Type** | **Participant Type** | **CFQ Type** | **Measure(s) included** |
| --- | --- | --- | --- | --- |
| Adjorlolo et al. (2016) | 1 | 18 | 1 | Stroop interference |
| Baker et al. (2018) | 1 | 16 | 1 | Stroop accuracy |
| Bellens et al. (2022) | 1 | 7 | 1 | Go/No-Go accuracy |
| Berende et al. (2019) | 1 | 32 | 6 | Stroop interference RT |
| Costa et al. (2023) | 2 | 2 | 2 | Stroop incongruent RT |
| Dey et al. (2019) | 1 | 35 | 1 | Stroop interference RT |
| Foster & Lavie (2007) | 1 | 1 | 1 | Flanker interference RT |
| Friedman & Miyake (2004) | 1 | 1 | 1 | Antisaccade Accuracy, Stop Signal RT, Stroop effect RT, flanker effect RT, word naming interference RT, shape naming interference RT |
| Gajewski & Falkenstein (2012) | 1 | 2 | 1 | Stroop interference |
| Gajewski & Falkenstein (2014) | 1 | 26 | 1 | Stroop interference |
| Gajewski & Falkenstein (2015) | 1 | 2 | 1 | Stroop interference |
| Gajewski et al. (2011) | 1 | 2 | 1 | Stroop interference |
| Gajewski et al. (2020) | 1 | 2 | 1 | Stroop interference RT |
| Gajewski et al. (2022) | 1 | 6 | 1 | Stroop interference |
| Gokal et al. (2018) | 1 | 17 | 1 | Stroop Interference RT |
| Gunduz et al. (2022) | 1 | 1 | 1 | Stroop Interference, flanker Interference |
| Gustavson et al. (2015) | 1 | 30 | 1 | Antisaccade Accuracy, Stroop RT Slowing, Stop Signal RT |
| Hagen et al. (2020) | 1 | 29 | 1 | Stroop (CWI-III) |
| Horvat & Tement (2020) | 1 | 1 | 1 | Flanker Accuracy, Flanker RT, Go/No-Go accuracy |
| Hsieh et al. (2022) | 1 | 9 | 1 | Go/No-Go accuracy |
| Ishigami & Klein (2009) Exp1 | 1 | 1 | 1 | Flanker RT (from ANT) |
| Ishigami & Klein (2009) Exp2 | 1 | 1 | 1 | Flanker RT (from ANT) |
| Jozwiak et al. (2017) | 1 | 38 | 1 | Stroop interference RT |
| Judah et al. (2014) - Study 4 | 1 | 1 | 1 | Antisaccade Accuracy |
| Kalpidou et al. (2022) | 1 | 1 | 1 | Flanker Accuracy, CPT accuracy |
| Keilp et al. (2013) | 1 | 33 | 1 | Stroop interference (RT) |
| Könen & Karbach (2020) | 1 | 6 | 4 | Flanker interference RT, Simon interference RT (combined by authors) |
| Krabbe et al. (2017) controls | 1 | 6 | 1 | Go/No-Go commission errors |
| Krabbe et al. (2017) stress-related exhaustion patients | 1 | 37 | 1 | Go/No-Go commission errors |
| Kramer et al. (1994) | 1 | 26 | 1 | Flanker interference RT, Stop Signal RT |
| Kreitz et al. (2015) - Study 1 | 1 | 1 | 4 | Flanker Interference (RT%increase) |
| Kreitz et al. (2015) - Study 2 | 1 | 1 | 4 | Flanker Interference (RT%increase) |
| Lopresti et al. (2022) | 1 | 11 | 1 | Stroop percent correct |
| Lopresti et al. (2023) | 1 | 10 | 1 | Stroop percent correct |
| Polsinelli et al. (2020) | 1 | 2 | 1 | Simon Incongruency-Proportional-to-Congruent (RT) |
| Ryskin et al. (2015) | 1 | 1 | 1 | Stroop Incongruent Accuracy |
| Slagboom et al. (2021) | 1 | 24 | 1 | Affective Go/No-Go Commission Errors |
| Slana & Repovs (2020) | 1 | 6 | 1 | Stroop |
| Swick & Ashley (2020) | 1 | 27 | 1 | Go/No-Go Error, Stop Signal RT |
| Tipper & Baylis (1987) | 1 | 6 | 1 | Incongruent Word Interference |
| Vom Hofe et al. (1998) | 1 | 1 | 1 | Stroop (RT) |
| Welhaf & Kane (2023) | 1 | 1 | 3 | Antisaccade accuracy, Stroop interference |
| Woltering et al. (2013) | 1 | 20 | 1 | No-Go Accuracy |
| Woods et al. (2024) | 1 | 6 | 1 | Stroop incompatible accuracy and RT |

**Category 4 (SART)**

| **Reference** | **Effect Size Type** | **Participant Type** | **CFQ Type** | **Measure(s) included** |
| --- | --- | --- | --- | --- |
| Bloomfield et al. (2010) | 1 | 18 | 1 | SART CE |
| Dockree et al. (2006) - controls | 1 | 6 | 1 | SART CE |
| Dockree et al. (2006) - TBI patients | 1 | 18 | 1 | SART CE |
| Donohoe et al. (2009) | 1 | 23 | 1 | SART CE |
| Farrin et al. (2003) | 1 | 14 | 1 | SART CE |
| Gokal et al. (2018) | 1 | 17 | 1 | SART CE |
| Koso et al. (2012) | 2 | 34 | 1 | SART CE |
| Larue et al. (2010) | 1 | 1 | 25 | SART accuracy |
| Manly et al. (1999) | 3 | 6 | 1 | SART CE |
| McVay & Kane (2009) | 1 | 1 | 3 | SART (d') |
| Righi et al. (2009) | 1 | 1 | 1 | SART CE |
| Robertson et al. (1997) | 1 | 6 | 1 | SART performance |
| Smilek et al. (2010) Study 2 | 1 | 6 | 1 | SART CE |
| van Der Linden et al (2005) | 1 | 21 | 1 | SART CE |
| Wallace et al. (2001) | 2 | 1 | 8 | SART CE |
| Whyte et al. (2006) - controls | 1 | 6 | 1 | SART CE |
| Whyte et al. (2006) - TBI patients | 1 | 18 | 1 | SART CE |
| Zanesco et al. (2019) | 1 | 14 | 1 | SART A' |

*Note.* CE = commission errors.

***Nominal Label Coding:***

- Participant Group
  - 1 = students
  - 2 = healthy older adults (at least 50+ years)
  - 3 = bilateral vestibular loss patients
  - 4 = unilateral vestibular loss patients
  - 5 = long covid patients
  - 6 = general community adults
  - 7 = breast cancer survivors with cognitive complaints
  - 8 = work-related stress patients
  - 9 = High & low trait anxiety groups from student population
  - 10 = older adults (60+) with mild or subjective cognitive impairment
  - 11 = 40-75 with self-reported subjective cognitive issues
  - 12 = previously hospitalised from COVID-19 infection
  - 13 = adults with autism
  - 14 = military personnel
  - 15 = HIV infected patients
  - 16 = patients with chronic back pain
  - 17 = breast cancer patients
  - 18 = traumatic brain injury patients (TBI)
  - 19 = cardiac arrest survivors
  - 20 = students with ADHD
  - 21 = work burnout & controls
  - 22 = older adults with amnesic MCI
  - 23 = patients with SCZ
  - 24 = Patients with hypopituitarism
  - 25 = MDMA users and non-users
  - 26 = younger and older adults
  - 27 = combat vets with PTSD and age-matched controls
  - 28 = stroke survivors
  - 29 = participants with MDD
  - 30 = twins
  - 31 = patients with testicular cancer and controls
  - 32 = patients with persistent symptoms attributed to Lyme disease
  - 33 = participants with MDD and controls
  - 34 = combat vets with PTSD
  - 35 = older adults with vascular risk factors
  - 36 = people with tinnitus
  - 37 = stress-related exhaustion patients
  - 38 = Parkinson’s patients & controls
- CFQ type
  - 1 = full original scale
  - 2 = memory subscale (8 items)
  - 3 = memory & attention lapses – 40 items
  - 4 = 32 item German version
  - 5 = Italian CFQ (25 items)
  - 6 = Dutch version
  - 7 = regression scores from 5 factors
  - 8 = regression scores from 4 factors
- Effect size type
  - 1 = bivariate Correlation
  - 2 = beta coefficient or partial correlation
  - 3 = F value for high and low CFQ groups

**Forest Plots Following Outlier Exclusion**

**Category 1 (Switching):**


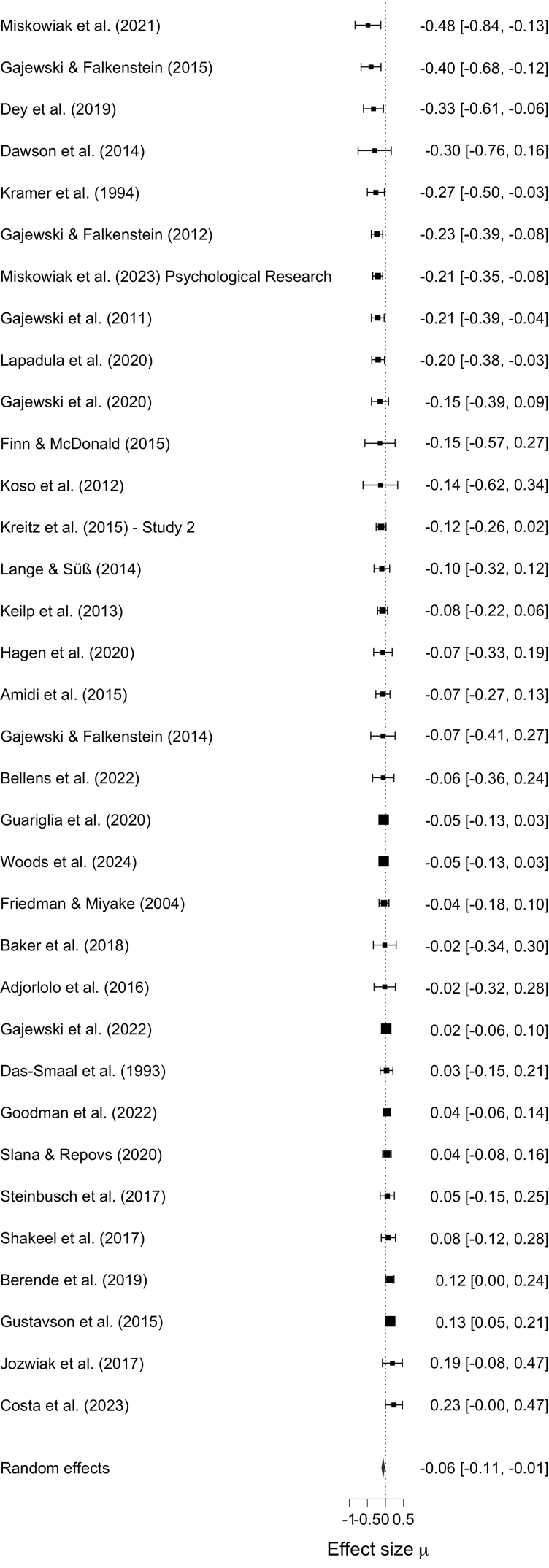


**Category 2 (Updating Working Memory):**


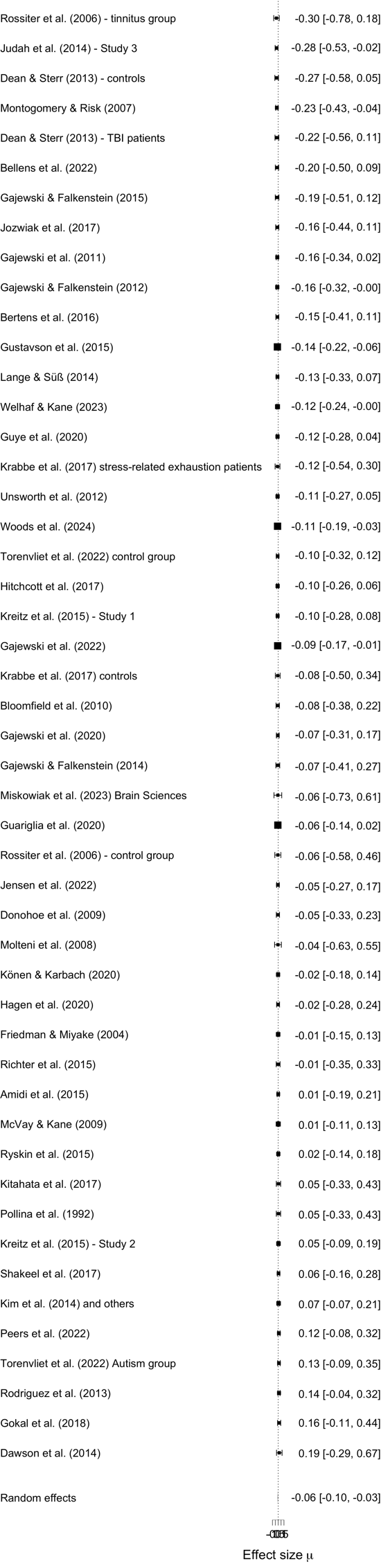


**Category 3 (Inhibition):**


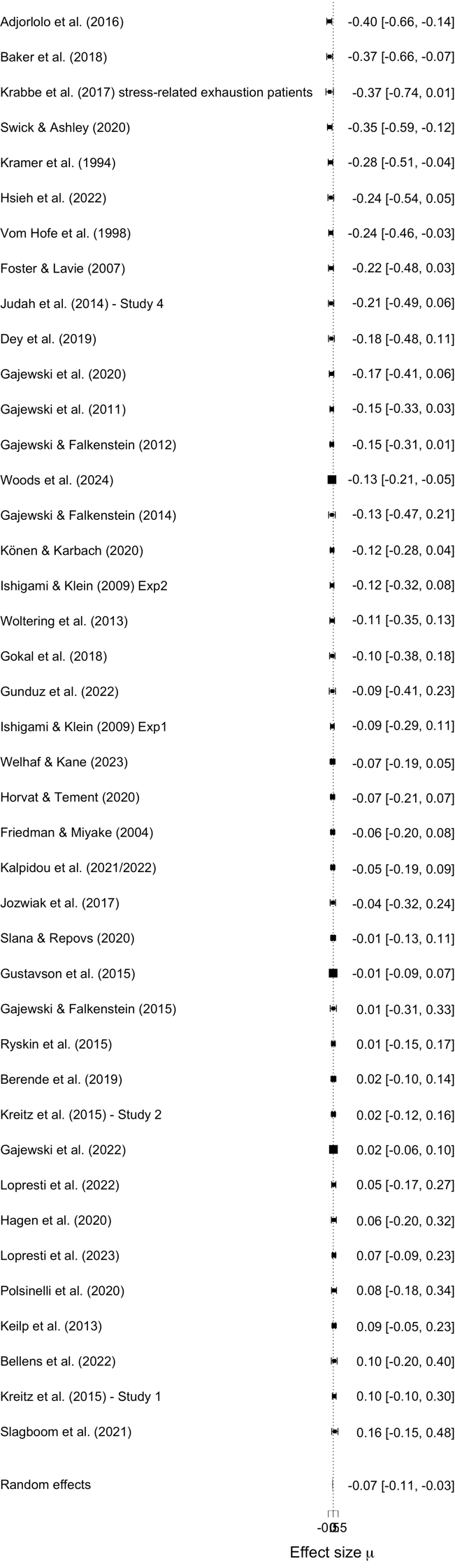


**Category 4 (SART):**


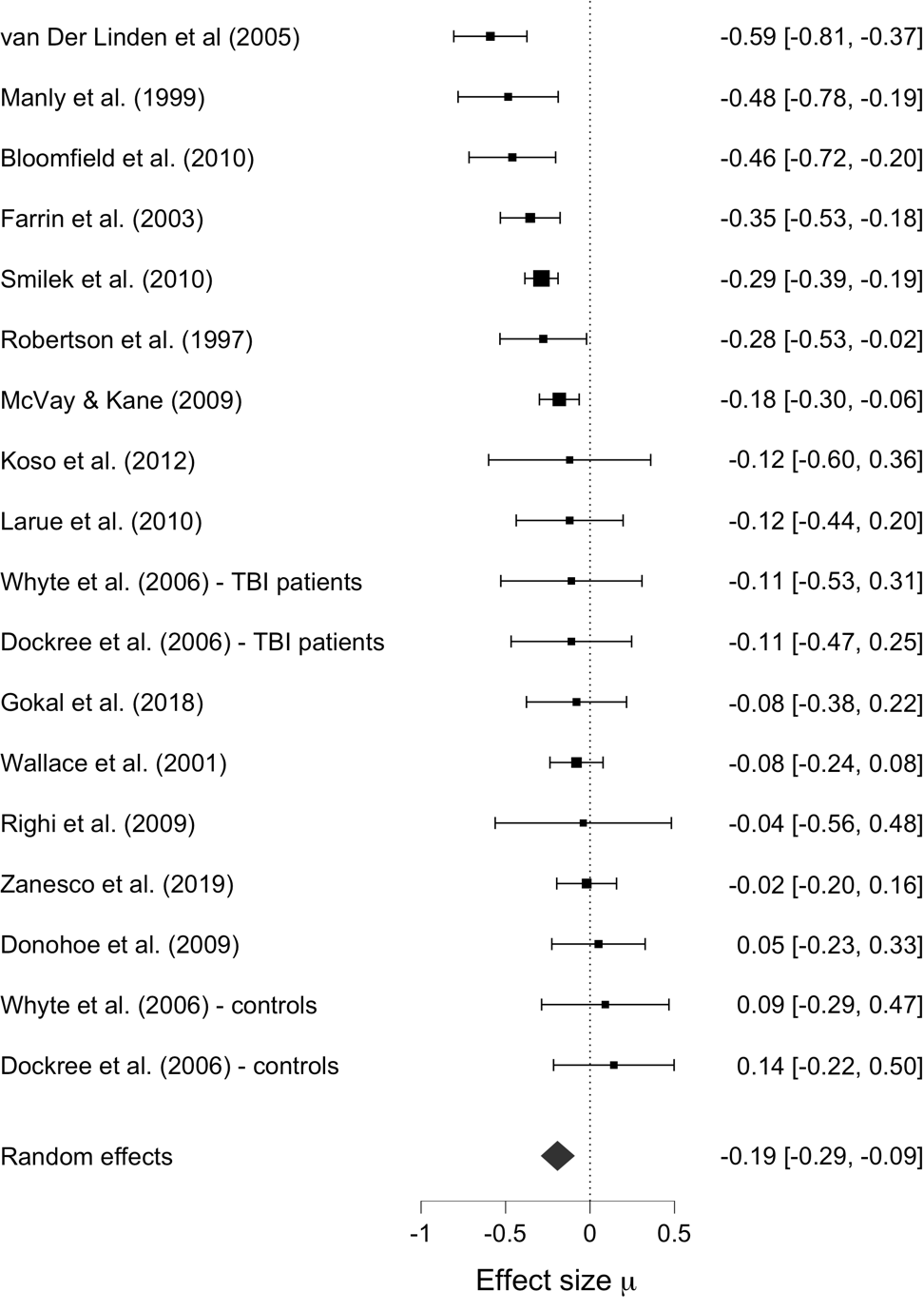

Supplement: Supplementary file 1 — Supplementary file1 (DOCX 1236 KB) [file 13423_2024_2573_MOESM1_ESM.docx]
